# High-performance ternary logic circuits and neural networks based on carbon nanotube source-gating transistors

**DOI:** 10.1126/sciadv.adt1909

**Published:** 2025-01-10

**Authors:** Xuehao Zhu, Meiqi Xi, Jianyu Wang, Panpan Zhang, Yi Li, Xiao Luo, Lan Bai, Xingxing Chen, Lian-mao Peng, Yu Cao, Qiliang Li, Xuelei Liang

**Affiliations:** ^1^ Key Laboratory for the Physics and Chemistry of Nanodevices and Center for Carbon-Based Electronics, School of Electronics, Peking University, Beijing 100871, China.; ^2^ Institute for Carbon-Based Thin Film Electronics, Peking University, Shanxi (ICTFE-PKU), Taiyuan 030012, China.; ^3^ Department of Advanced Manufacturing and Robotics, College of Engineering, Peking University, Beijing 100871, China.; ^4^ State Key Laboratory of Information Photonics and Optical Communications, Beijing University of Post and Telecommunications, Beijing 100876, China.; ^5^ Academy for Advanced Interdisciplinary Studies, Peking University, Beijing 100871, China.; ^6^ Institute of Advanced Functional Materials and Devices, Shanxi University, Taiyuan 030006, China.

## Abstract

Multi-valued logics (MVLs) offer higher information density, reduced circuit and interconnect complexity, lower power dissipation, and faster speed over conventional binary logic system. Recent advancement in MVL research, particularly with emerging low-dimensional materials, suggests that breakthroughs may be imminent if multistates transistors can be fabricated controllably for large-scale integration. Here, a concept of source-gating transistors (SGTs) is developed and realized using carbon nanotubes (CNTs). By extending the source electrode into the channel of conventional CNT transistors, a controllable p-n homojunction is formed, allowing CNT-SGTs to reliably switch between three distinct states. Capitalizing on the straightforward fabrication process of CNT-SGTs, ternary inverters, NMIN and NMAX logic gates, ternary SRAM cells, and a ternary neural network achieving 100% image classification accuracy have been successfully implemented. This study represents the most advanced and highest-performing ternary circuits realized with low-dimensional materials to date. This progress highlights the potential of CNT-SGTs in driving the future of MVL architectures.

## Teaser

Carbon nanotube source-gating transistors are developed for ternary logic computing architecture and ternary neural networks.

## INTRODUCTION

Silicon-based complementary metal-oxide semiconductor (CMOS) technology has driven high-performance, low-power chips at decreasing costs for over 50 years (*1*, *2*). However, as the semiconductor industry nears the physical limits of silicon CMOS, significant challenges are emerging (*3*). As transistors shrink to the nanoscale, quantum effects become more pronounced, complicating the maintenance of predictable electronic behavior. In addition, metal interconnects in modern chips, housing billions of transistors, occupy a large portion of chip area and consume more than 70% of the total dynamic power (*4*), creating a major bottleneck for energy-efficient computing systems. To address these challenges, new materials, devices, and computing architectures are required (*3*, *5*, *6*).

Emerging low-dimensional materials, such as two-dimensional (2D) materials (*7*, *8*) (e.g., graphene, 2D transition metal dichalcogenides, etc.) and 1D single-walled carbon nanotubes (CNTs) (*9*, *10*), offers promising pathways for improving device performance and efficiency. These materials can maintain high-quality crystal lattices and good mobility even at subnanometer thickness and enable new device concepts such as CNT-based Dirac source transistors (*11*) and 2D material-based van der Waals heterostructure devices (*7*, *8*). Moreover, beyond extending Moore’s law for binary logic integrated circuits (ICs), these emerging materials and devices present opportunities for multi-valued logic (MVL) systems, which use multiple states to represent more information per bit, enabling complex operations with fewer logic gates and interconnects and leading to lower power consumption and more efficient thermal management (*12*–*16*). MVL systems also benefit from artificial intelligence (AI), particularly in managing imprecision and ambiguity by enabling more complex computations with reduced memory requirements. For example, using ternary weights {−1, 0, +1} in training deep neural networks (DNNs) can notably reduce computational complexity, speed up inference processes, and decrease memory requirements by factors of approximately 16× or 32× compared to 32-bit or 64-bit precision (*17*, *18*).

Despite the advantages, binary logic has traditionally prevailed due to the difficulty of creating devices with reliable and distinct states beyond two. Recent advancements in MVL devices and structures based on emerging nanomaterials indicate that MVL is gaining momentum, but most of the reported MVL devices are fabricated using heterostructures of 2D materials that leverage negative differential transconductance (NDT) or negative differential resistance (NDR) effects arising from p-n junctions within these structures (*14*). Although achieving multiple logic states, their fabrication process remains complex due to the need for exfoliation and transfer of different 2D semiconducting materials and precise placement of heterojunctions. This complexity limits scalability and poses notable challenges in mass production. Therefore, developing a straightforward, scalable fabrication method for MVL technology that ensures precise positioning of p-n junctions within the device channel is crucial.

In this paper, we propose a source-gating transistor (SGT) design for MVL based on single-walled CNTs. In this design, the source electrode extends into the channel, creating a homo-structured p-n junction with a precisely controlled location. Prominent NDT/NDR effects with a high output current peak-to-valley ratio (PVR) were observed. The NDT mechanism, attributed to band-to-band tunneling (BTBT) at the p-n junction, was confirmed by technology computer aided design (TCAD) simulation. Using these CNT-SGTs, we fabricated ternary logic inverters with “rail-to-rail” voltage transfer curves (VTCs) and high voltage gains. Building on these ternary inverters, we implemented more complex ternary logic circuits, including NMIN, NMAX, and ternary six-transistor static random-access memory (ternary 6 T-SRAM) circuits. In addition, we developed a ternary neuron (TN) circuit comprising ternary logic gates, laying the basis for ternary processing and computation. Using these artificial TNs, we constructed and analyzed a ternary neural network (TNN) consisting of 8 × 8 TNs, which achieved exceptional accuracy in recognizing handwritten digits.

## RESULTS

### Design and properties of CNT-SGTs

**Fig. 1.**
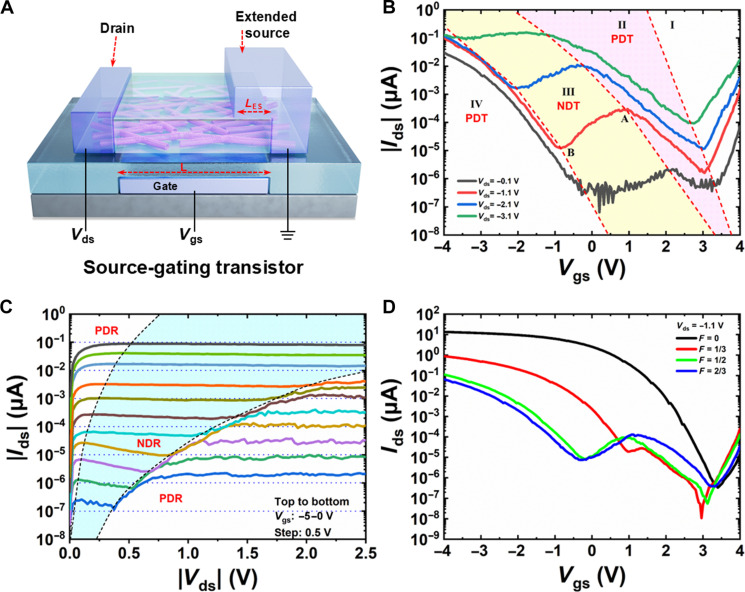
Performance of a typical CNT-SGT. (**A**) Schematic of CNT-SGT’s structure. (**B**) Transfer and (**C**) output characteristics of a typical CNT-SGT with *W* = 10 μm, *L* = 5 μm, and *F* = 2/3. (**D**) Transfer characteristic curves of CNT-SGTs with same *W* of 10 μm and *L* of 5 μm but various *F*. *F* = 0 corresponds to a conventional CNT transistor. The devices were measured using negative *V*_ds_, but the absolute values of the output current were plotted in semi-logarithmic scale in (B) to (D).

We propose a three-terminal CNT transistor for MVL, as depicted in Fig. 1A. In this design, a bottom gate spans across the source and drain electrodes, modulating the energy band of the entire channel similar to conventional CNT transistors. A unique feature of this device is the extended source electrode, which covers part of the channel. Unlike conventional transistors where the source electrode is grounded and merely functions as a carrier injection point, the extended source in our device also acts as an active gate with its electrostatic potential fixed at zero voltage. Thus, we term this structure a CNT-SGT. The extended source reduces the coupling between the channel and the bottom gate, resulting in varying tuning efficiencies across the channel. This variation enables the formation of a homo-structured p-n junction during bottom gate sweeping, leading to observable NDT phenomena that facilitate MVL. Our design simplifies fabrication compared to 2D heterostructures (*14*) and avoids the circuits’ complexity of a four-terminal configuration with two independent gate electrodes in conventional CNT devices (*19*, *20*). Although the CNT-SGTs use a bottom-gate structure, a top-gate configuration is also feasible by inverting the positions of the gate and the extended source. Detailed procedures for CNT material preparation, characterization and device fabrication are provided in Materials and Methods (also see figs. S1 and S2).

The effectiveness of this design was validated through measurements shown in Fig. 1 (also see figs. S3 to S5). Figure 1B presents the transfer curves of a device with a channel length (*L*) of 5 μm and an extended source length (*L*_ES_) of 3.3 μm. These transfer curves exhibit a characteristic “W” shape as the gate voltage (*V*_gs_) is varied from negative to positive values, divided into four regions (I to IV). Compared to a conventional CNT transistor with the same *L* and source/drain contacts (fig. S3), the current increase in region I (*V*_gs_ > 3 V) is attributed to the ambipolar behavior of CNT devices, specifically the activation of the n-branch due to electron tunneling from the drain side (*21*). Regions II and IV show a decreasing trend in current with increasing *V*_gs_, i.e., positive differential transconductance consistent with the p-branch behavior of conventional CNT transistors, where the current decreases as the transistor turns off. In contrast, region III shows an increase in current with *V*_gs_, demonstrating NDT effects and a peak current with a PVR of 25 (fig. S4). The observed NDT effects and three distinct states (regions II, III, and IV) result from the co-coupling of the gate electrode and the extended source electrode to the channel, within the voltage range corresponding to the p-branch of a conventional CNT transistor. The NDT effect is evident even at a low source-drain bias (*V*_ds_) of 0.1 V, as shown in Fig. 1B, indicating its practicality in CNT-SGTs. As *V*_ds_ increases, the NDT effect and current peak become more pronounced but eventually diminish at higher *V*_ds_ levels, while the three distinct states remain distinguishable. Given that the NDT phenomena occur within the p-branch voltage range of a typical CNT transistor, this device is classified as a p-type CNT-SGT. Development of n-type CNT-SGTs is anticipated and is part of our future research plans.

The NDT observed in the CNT-SGTs is due to BTBT across the p-n junction in the channel, which can also induce NDR effects (*22*). These effects were also observed in the output curves of the CNT-SGT, as shown in Fig. 1C (also see fig. S5). At lower *V*_gs_, the output current rapidly increases with initial *V*_ds_ but then decreases after reaching a peak, showing NDR behavior. As *V*_ds_ increases further, the current rises again, displaying positive differential resistance (PDR) and creating a kink in the output curve. With increasing *V*_gs_, the NDR region expands and the kink between the NDR and PDR regions diminishes. This suggests a competing coupling between *V*_gs_ and *V*_ds_ in modulating the channel potential.

The extended source in the CNT-SGT divides the channel into two parts, each tuned with different efficiencies by the bottom gate. The ratio of the extended source length to the total channel length, defined as *F* = *L*_ES_/*L*, notably affects the device performance, as illustrated in Fig. 1D for devices with identical *L* but varying *L*_ES_. As *F* increases from 0 to ^1^/_3_, the device transits from a conventional CNT transistor to a CNT-SGT, characterized by a small current peak during *V*_gs_ sweep, indicative of a weak NDT effect. When *F* increases to one-half and two-third, the NDT effect becomes more pronounced, and the voltage range of the NDT region expands. Consequently, the maximum output current (measured at *V*_gs_ = −4 V) decreases with increasing *F*. Thus, optimizing *F* can enhance the performance of CNT-SGTs.

**Fig. 2.**
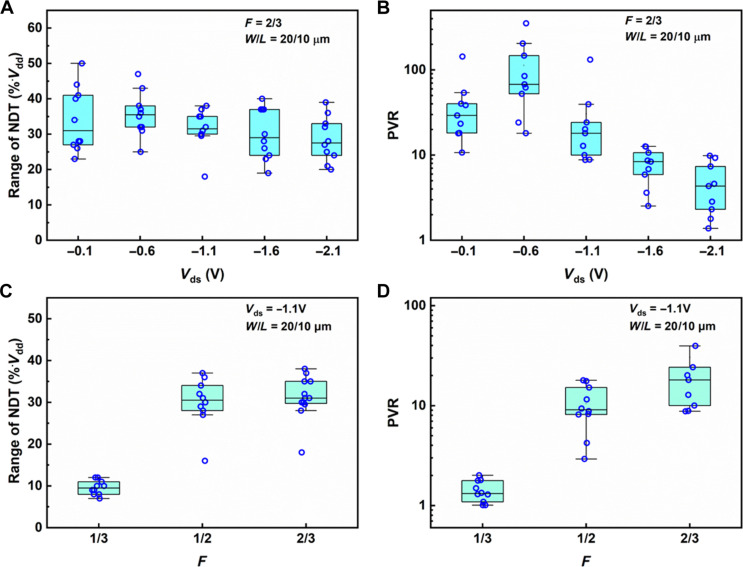
Influence of *V*_ds_ and *F* on the properties of CNT-SGTs (**A** and **B**) Influence of *V*_ds_ on the range of NDT (A) and PVR (B), respectively. (**C** and **D**) Influence of F on the range of NDT (C) and PVR (D), respectively. All devices were measured with *V*_gs_ sweep range of 0 to −4 V.

The NDT effects depicted in Fig. 1 are not isolated incidents limited to individual devices, but are observed across nearly all fabricated CNT-SGTs, as illustrated by the statistical results shown in Fig. 2. More representative transfer curves are also provided in fig. S6. Figure 2 (A and B) illustrates the influence of *V*_ds_. For the representative case of *F* = 2/3, which typically exhibits pronounced NDT, the voltage range where NDT occurs (i.e., the width of region III in Fig. 1B) slightly decreases with increasing *V*_ds_. However, the NDT range still spans a significant proportion (about 30 to 40%) of the total gate voltage, ensuring that the three states (positive-negative-positive transconductance) are observed with comparable frequency. This characteristic meets the requirements for devices intended for ternary logic applications (*13*). The PVR values of the NDT effects, shown in Fig. 2B, can reach several hundreds (also see fig. S4). These values are notably high compared to those reported for 2D heterostructure devices (see table S1). Figure 2 (C and D) illustrates the influences of *F* on the NDT range and PVR, respectively, with both metrics increasing with *F*, consistent with the results shown in Fig. 1D.

**Fig. 3.**
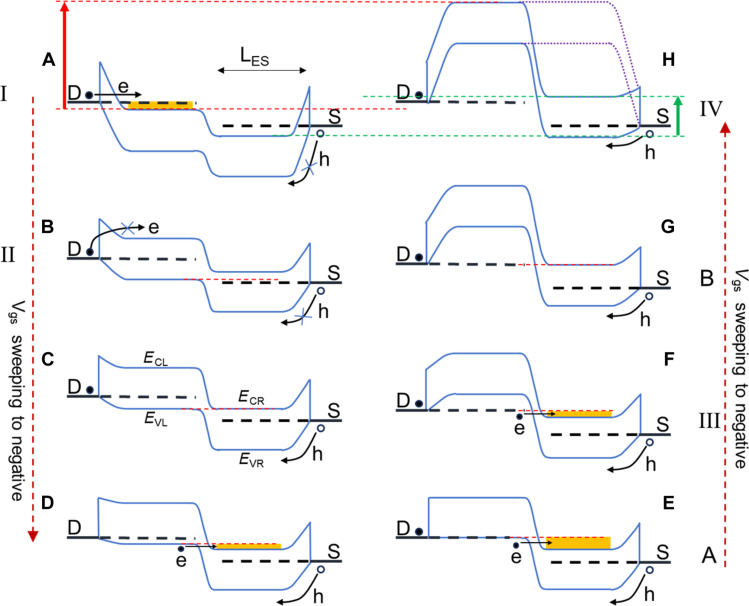
Energy-band diagram of the CNT-SGT operation. (**A** to **H**) Energy-band (solid blue lines) diagram of CNT-SGT with *V*_gs_ sweeping from positive to negative. The purple dotted lines in (H) represent energy band of a conventional CNT transistors. The energy band of the channel outside the extended source (left part of the channel) is tuned more efficiently. Labels of I to IV, A and B correspond to those in Fig. 1B.

Next, we systematically investigate the mechanism behind the NDT effects. As expected, the gate modulation of the energy band in the channel region beneath the extended source lags behind the uncovered region due to the capacitive coupling with the extended source. In addition, the source-gating region creates a conformal barrier at the source end, preventing holes from tunneling into the channel; thus, holes can only be injected into the channel via thermal emission. Figure 3 qualitatively depicts the energy band modulation during a *V*_gs_ sweep from positive to negative values in the CNT-SGT. Figure 3A shows the energy band structure under a very positive *V*_gs_, corresponding to region I in Fig. 1B. The positive *V*_gs_ pulls the entire energy band down, with the region under the extended source (right part of the channel) being pulled lower. Electrons can tunnel into the channel through the thin barrier at the drain end, while holes cannot effectively enter the channel at the source, leading to the previously discussed ambipolar behavior. As *V*_gs_ decreases, the energy band rises and the barrier at the drain end thickens, which hinders electron tunneling, while the barrier for holes injection from the source end remains very high. Consequently, the current tends to switch off as *V*_gs_ decreases, as shown in Fig. 3B. Without source gating, the energy band of the left channel part is tuned more effectively than the region beneath the source-gating area, causing it to rise more quickly as *V*_gs_ becomes more negative. This leads to the formation of a p-n junction, as depicted in Fig. 3C. When the valence band edge of the left part (*E*_VL_) aligns with the conduction band edge of the right part (*E*_CR_), a band overlap region forms, triggering band-to-band tunneling (BTBT) of electrons. Although holes injection at the source end remains negligible, the total current increases due to BTBT (Fig. 3D). When the *E*_VL_ aligns with the Fermi-level of the drain, the electron tunneling current reaches its maximum as the available unoccupied states in the n-side increase with *V*_gs_, corresponding to point A in Fig. 1B, as shown in Fig. 3E. Beyond this point, available unoccupied states in the n-side decrease, suppressing the electron tunneling. Although the thermal injection of holes may increase due to the lowering of the barrier at the source end, it cannot fully compensate for the decreased tunneling current, leading to a reduction in total current as shown in Fig. 3F, corresponding to region III of Fig. 1B. When *E*_CR_ aligns with the Fermi level of the drain, as shown in Fig. 3G, the current drops to a minimum (point B in Fig. 1B) as available states for tunneling vanish. As *V*_gs_ decreases further, the barrier at the source end becomes low enough for holes to overcome it easily. This causes a rapid increase in hole current, which then dominates the total current, making the onset of the p-type branch typical of conventional CNT transistors, as shown in Fig. 3H. The on-state output current is primarily determined by the band structure in the right channel region. Compared to conventional CNT transistors, the energy band in right channel of the CNT-SGT is lower due to coupling with the extended source, resulting in a reduced hole concentration and, consequently, a lower output current, as shown in Fig. 1D. Increasing *V*_gs_ further raises the energy band in the right channel, which in turn enhances the output current. In addition, using an aligned CNT array, instead of the network film, boosts the output current. Although the current is lower than in conventional CNT transistors, it exceeds that of most reported MVL devices based on 2D materials (*23*–*27*), highlighting the advantages of our CNT-SGTs.

To further understand the CNT-SGT design, we performed TCAD simulations using Sentaurus (simulation in Supplementary Text). The TCAD simulations confirmed the occurrence of NDT phenomena, demonstrating the increase and decrease of the interband tunneling effect inside the channel, as shown by the Band2BandGeneneration plots in fig. S7 (A and B). Systematic TCAD simulations were performed to investigate the scalability of our design. The results indicate that the CNT-SGTs maintain remarkable NDT characteristics even when scaled down to 200 and 50 nm, with the extended source covering half of the channel length, as depicted in fig. S7 (C and D). This highlights the considerable miniaturization potential of CNT-SGTs, provided that the p-n homojuncion can be effectively engineered. Notably, although the SGT structure is also asymmetric between the source and drain, its working mechanism differs from previously reported devices with asymmetric designs at the drain end (*28*–*30*). Those designs aim to suppress minority carrier tunneling from the drain electrode into the channel and enhance the on/off ratio, such as suppressing current in region I of Fig. 1B. In contrast, our asymmetric design at the source end is intended to create a p-n junction within the channel and initiate inter-band tunneling.

### Ternary logic circuits based on CNT-SGTs

**Fig. 4.**
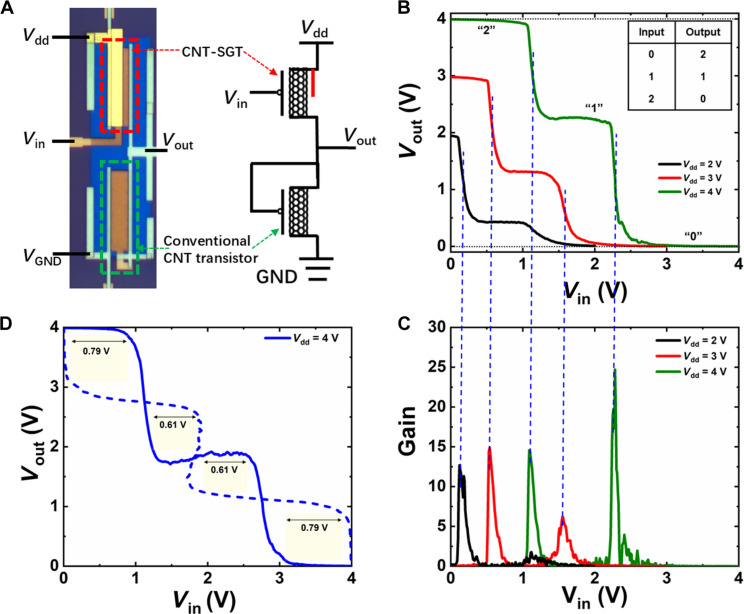
Performance of ternary inverters based on CNT-SGTs. (**A**) Optical image and circuit diagram of a ternary inverter. *W*/*L* = 20 μm/5 μm, *F* = 2/3 for the CNT-SGT, and *W*/*L* = 20 μm/5 μm for the conventional CNT transistor. Corresponding VTCs (**B**) and voltage gains (**C**) of this ternary inverter. (**D**) SNM extracted from a ternary inverter consists of CNT-SGT with *F* = 1/2.

Confident that the CNT-SGTs meet the primary requirement of having three distinct stable states for ternary logic building blocks, we proceeded to fabricate ternary logic circuits. Figure 4A shows the optical microscope image and the circuit diagram of a ternary inverter, the fundamental unit for ternary logic circuits. In this configuration, a CNT-SGT is connected in series with a conventional CNT transistor. Because n-type CNT-SGTs face similar challenges in achieving robust n-type behavior compared to conventional n-type CNT transistors, a conventional p-type CNT transistor is used as an active load, with its gate electrode connected to the inverter’s output. Using the p-type CNT-SGT transistor, the inverter was constructed in a pure PMOS style. Figure 4B presents the typical VTCs of this inverter, demonstrating nearly rail-to-rail outputs with three distinct voltage levels corresponding to logic states “2,” “1,” and “0.” The nearly symmetric VTC behavior of the ternary inverter is explained by the properties of its components, detailed in fig. S8. In this PMOS configuration, the conductance of the load transistor remains nearly constant. At low input voltage (*V*_in_) levels (*V*_gs_ < −2.5 V in fig. S8), the conductance of the CNT-SGT is much higher than that of the conventional load transistor, causing most of *V*_dd_ to drop across the load and resulting in a high *V*_out_ level close to the supply voltage (*V*_dd_). At high *V*_in_ levels (*V*_gs_ > −0.6 V in fig. S8), the conductance of the CNT-SGT is substantially lower than that of the load, leading to a *V*_out_ close to 0 V. In the intermediate range of *V*_in_ (−2.5 V < *V*_gs_ < −0.6 V in fig. S8), the relatively small PVR value of the CNT-SGTs results in a small variation in the conductance ratio between the two components. Therefore, *V*_dd_ is almost equally shared, producing a stable middle logic level in the PMOS-style ternary inverter (*31*–*33*). The flatness of the middle logic step could be improved using step-like NDT phenomena, as shown by the curve of *V*_ds_ = −3.1 V in Fig. 1B. Alternatively, a CMOS-style ternary inverter design using an n-type conventional CNT transistor as the load (*34*, *35*), which may have a transfer curve parallel to that of the CNT-SGT in the NDT region, could achieve a very flat middle logic state. This is an area of ongoing research. The inverter exhibits fast switching due to gate modulation of the CNT-SGT transistor, resulting in high voltage gains for transitions between three logic states, as shown in Fig. 4C. The voltage gain increases with *V*_dd_. At *V*_dd_ = 4 V, the gain for transitions from logic state 2 to 1 (gain-1) reaches 15, while the gain for transition from 1 to 0 (gain-2) reaches 25. The *V*_out_ of the middle logic states as shown in Fig. 4B is very close to 1/2 *V*_dd_, particularly for *V*_dd_ = 3 and 4 V, which is crucial for the noise margin and scalability of ternary logic circuits. By mirroring the measured VTC, a butterfly plot was obtained, as shown in Fig. 4D (also see fig. S9), and the static noise margin (SNM) of the inverter was extracted by nesting the largest possible square within the plot. The SNM obtained for the inverter shown in Fig. 4D is 0.61 V, approximately 61% of the ideal value (1/4 *V*_dd_).

**Fig. 5.**
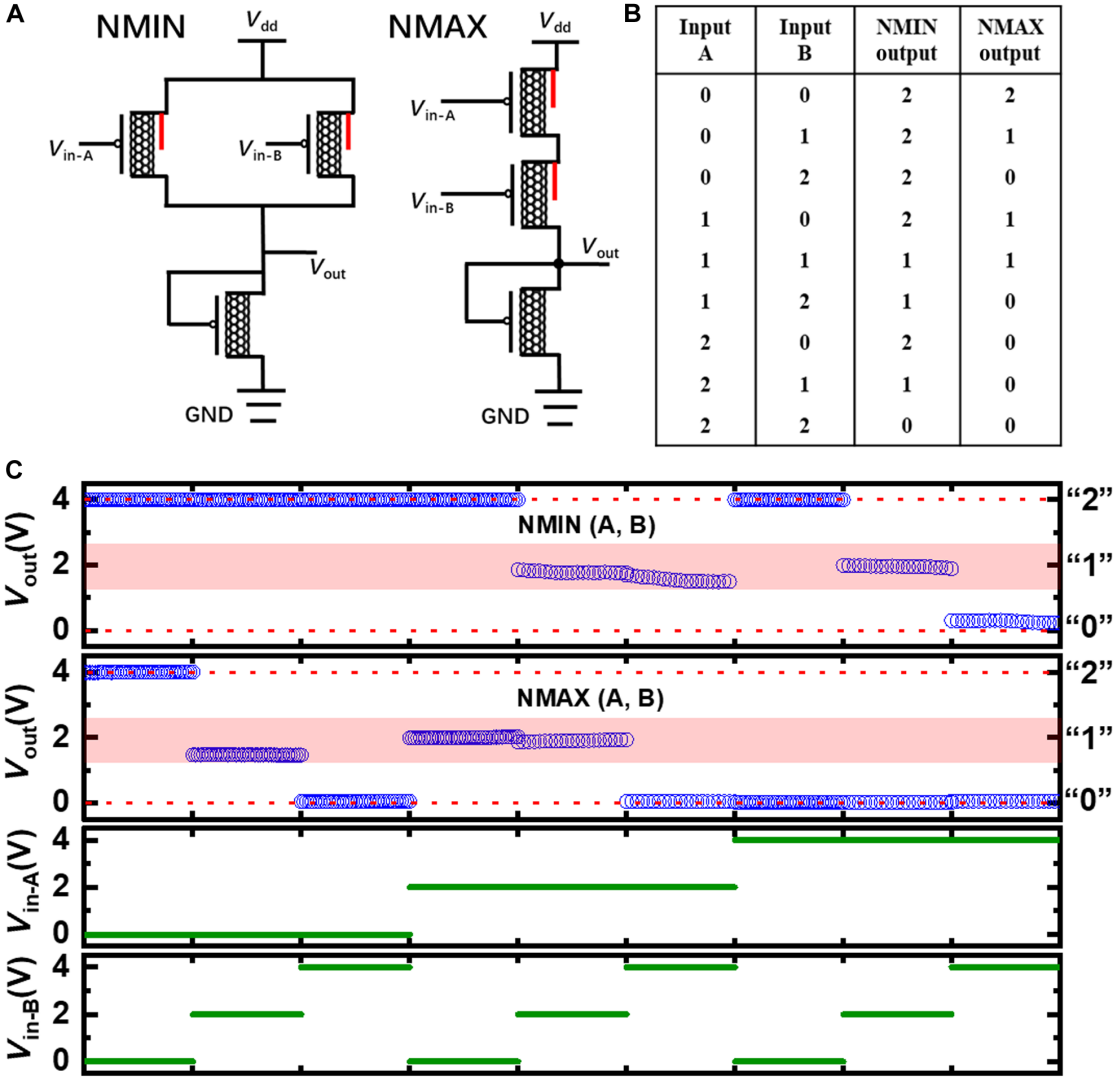
Performance of ternary NMIN and NMAX circuits. (**A**) Circuit diagrams and (**B**) corresponding truth table of ternary NMIN and NMAX. (**C**) Output characteristics of ternary NMIN and NMAX with varying inputs (A, B).

The rail-to-rail VTC with large SNM of the high-performance ternary inverters allows the development of other fundamental ternary logic gates and more complex circuits. In MVL, NMIN, and NMAX logic gates are crucial, replacing the common NAND and NOR gates in binary logic systems (*12*, *13*). The NMIN gate provides the inverse of the lesser input value, while the NMAX gate gives the inverse of the greater input value. Figure 5A illustrates the circuit diagrams of ternary NMIN and NMAX gates with their corresponding optical images shown in fig. S10. Structurally, the NMIN and NMAX gates are similar to binary NAND and NOR gates in a PMOS configuration, featuring two input terminals and a single output terminal. The key difference is the use of CNT-SGTs instead of traditional transistors. Figure 5B presents the logic truth table for the NMIN and NMAX gates, and Fig. 5C displays the corresponding measurement results. With *V*_dd_ of 4 V, the output logic value 2 precisely matches 4 V, indicating no voltage loss. The logic 0 level slightly deviates from 0 V, with voltages less than 0.3 V (<7.5% of *V*_dd_) for the NMIN gate and less than 0.05 V (< 1.25% of V_dd_) for the NMAX gate. Although the output voltage for the intermediate logic level 1 deviates relatively more from the ideal 2 V (1/2 *V*_dd_), it remains distinguishable from the logic levels 2 and 0 well within the noise margin. Compared to literature results (*36*, *37*), the deviations here are significantly smaller. These results validate that our developed ternary logic gates are capable of performing their logical functions accurately, a testament to the rail-to-rail output capability and the substantial noise margin provided by the ternary inverters. Furthermore, the deviations from the 1/2 *V*_dd_ level are mainly due to impedance mismatches between the CNT-SGTs and the connected loads, which can be mitigated through device optimizations.

**Fig. 6.**
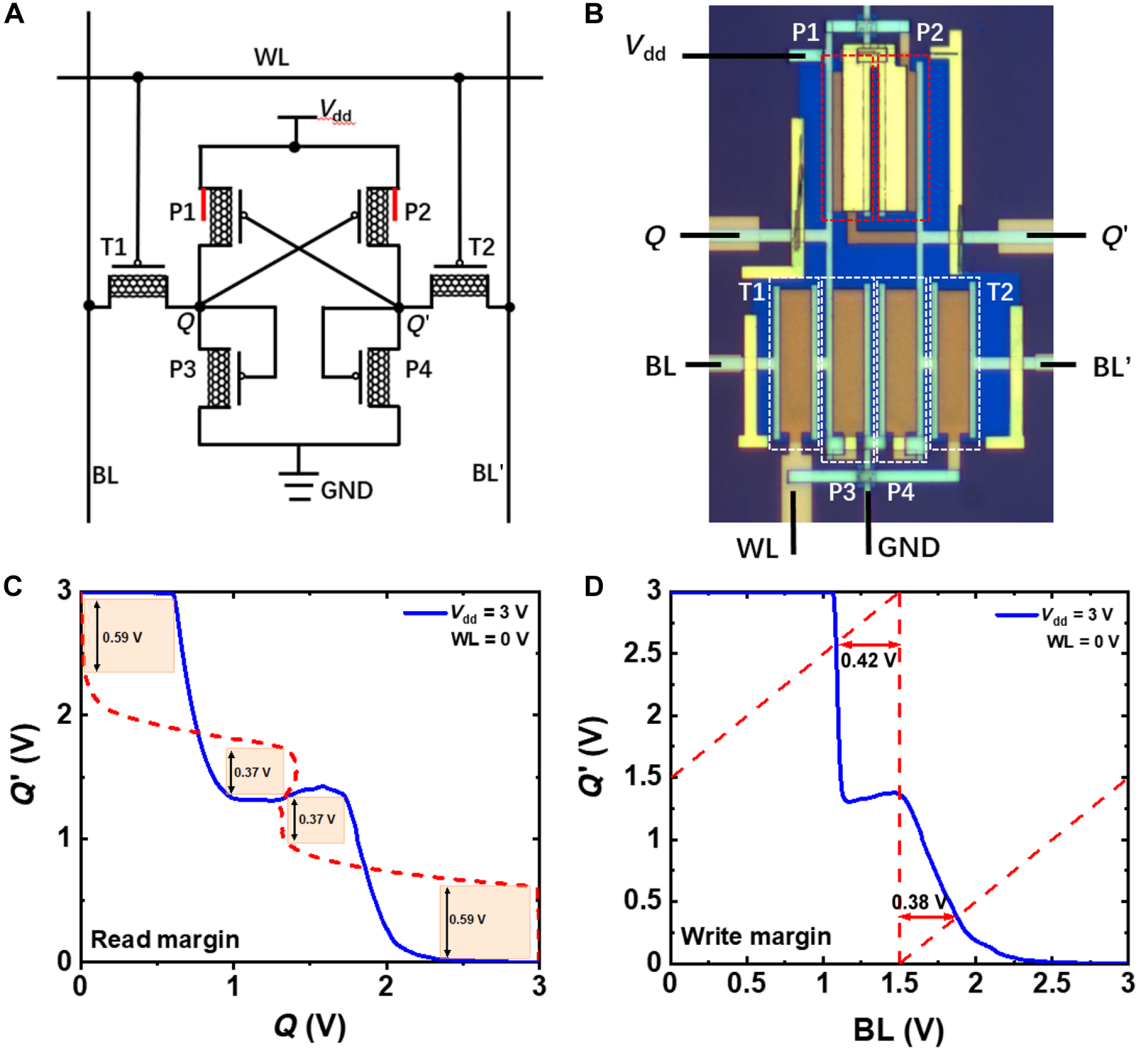
Performance of ternary 6 T-SRAM. (**A** and **B**) Circuit diagram (A) and optical image (B) of a ternary 6 T-SRAM circuit. The setup includes two CNT-SGTs (P1 and P2) paired with two conventional CNT transistors (P3 and P4) as loads. T1 and T2 are two access transistors for external communication. (**C** and **D**) Read (C) and write margin (D) of the ternary 6 T-SRAM cell.

Building on our success with the foundational logic gates, we now focus on developing more complex circuits such as a 6 T-SRAM cell, which is crucial for data storage. In a binary 6 T-SRAM cell, the core is a pair of cross-coupled binary inverters that form a latch with two stable states representing 0 and 1. Access transistors control the connection between the bit lines and the storage nodes via a word line, enabling read and write operations. During a read operation, the word line is activated to connect the storage nodes to the bit lines through the access transistors, allowing the data to be read. During a write operation, the bit lines are precharged to desired voltages, and the word line is activated to write data into the cell through the access transistors. For a ternary 6 T-SRAM cell, we replaced the binary SRAM core with a pair of cross-coupled PMOS-style ternary inverters for storing three stable states, 0, 1, and 2, as shown in Fig. 6 (A and B). Figure 6 (C and D) demonstrates the read and write margin of the ternary 6 T-SRAM (also see fig. S11), respectively. The SNM for reading is 0.37 V, nearly 50% of the ideal value. The write noise tolerance is 0.42 V for the transition from 2 to 1 and 0.38 V for the transition from 1 to 0.

### TNN based on CNT-SGTs

**Fig. 7.**
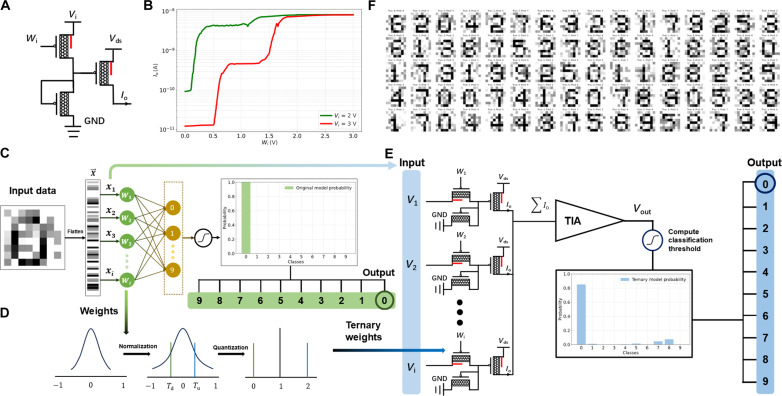
TN and neural network based on CNT-SGTs. (**A**) Diagram of the structure of a typical TN. (**B**) Simulated output characteristics for the TN with various weights and inputs. (**C**) A single-layer perceptron network with full-precision weights for classification of digits 0 to 9. (**D**) Normalization and quantization process to convert full-precision weights to ternary weights. (**E**) Diagram of the TNN consists of 8 × 8 TNs. (**F**) Recognition results of digits 0 to 9. Although the inputs are added with ±0.5 noise, the TNN yields a 100% accuracy rate.

MVL using CNT-SGTs shows promise for neural network applications. We designed and simulated TNs and a TNN based on the current-voltage characteristics of high-performance ternary inverters. Each TN, as illustrated in Fig. 7A, consists of a ternary inverter and an additional CNT-SGT, with the inverter’s output terminal connected to the additional CNT-SGT’s gate. The input voltage $V_{i}$ (where $V_{i}\in \left\{0,3\;V\right\}$) is fed into the neuron, and the weight factor $W_{i}$ results in three distinct conductance states. With $V_{\text{ds}}$ set to −2.1 V, $I_{o}$ represents the neuron’s output current. The simulated transfer characteristic curve of $I_{o}\;\text{versus}\;W_{i}$ based on experimental data is shown in Fig. 7B. A single-layer perceptron network as the base for the TNN was then implemented (Fig. 7C), processing noisy 8 × 8 pixel images of digits 0 to 9. The inputs $x_{1},x_{2},\ldots ,x_{i}$ are flattened and fed into 64 neurons, with forward propagation calculating original model probabilities and selecting the index with the highest probability as the output. The cross-entropy loss function is given by

$$\text{Loss}=-\sum_{i}y_{i}\text{log}\left(\hat{y_{i}}\right)$$ (1)

where $y_{i}$ is the true label and $\hat{y_{i}}$ is the predicted probability. During backpropagation, the weights $W_{1},W_{2},\ldots ,W_{i}$ are updated until the loss stabilizes after 100 epochs, resulting in original full-precision weights, which are then converted to ternary values {0,1,2} through normalization and quantization (Fig. 7D). The TNN circuit, depicted in Fig. 7E, uses an 8 × 8 array of TNs and weights. Inputs $x_{i}\in \left[0,1\right]$ are converted to $V_{i}\in \left\{0,3\;V\right\}$ using a threshold (*x*-th) of 0.5. Output currents $I_{o}$ are summed and fed into a transimpedance amplifier to obtain the classification voltage $V_{\text{out}}$. The index of the class threshold $T_{i}$ closest to $V_{\text{out}}$ is selected as the classification result. This process can be formalized as $\text{argmin}\left(|V_{\text{out}}-T_{i}|\right)$, where $T_{i}$ is

$$T_{i}=\frac{1}{N_{i}}\sum_{j=1}^{N}{\hat{Y}}_{\text{train},J}\cdot I\left(y_{\text{train},j}=i\right)$$ (2)

and $N_{i}$, the number of training samples in the i-th class, is

$$N_{i}=\sum_{j=1}^{N}I\left(y_{\text{train},j}=i\right)$$ (3)

${\hat{Y}}_{\text{train},J}$ is the model output for the j-th training sample, and $I\left(y_{\text{train},j}=i\right)$ is an indicator function that equals 1 if the j-th training sample belongs to the *i*-th class and 0 otherwise. Comparing the probability images in Fig. 7 (C and E), quantization results in decreased accuracy of the TNN compared to the original single-layer perceptron network, but the images remain accurately classified. In addition, our dataset includes 1500 training images and 300 test images, each class representing 1/10 of the total, with random noise in inputs (variation in $x_{i}$ = ±0.5). Figure 7F shows that the test accuracy reaches 100% when true and predicted values match, demonstrating the high potential of CNT-SGT MVL in TNN applications.

## DISCUSSION

**Fig. 8.**
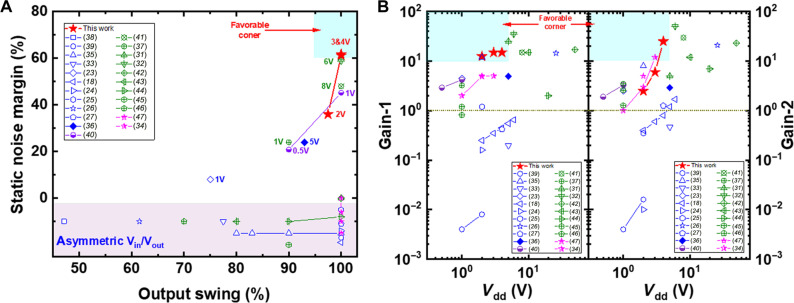
Benchmarking of ternary inverters. (**A**) Comparison of SNM value and output swing. The corresponding operating voltages are marked for the data of symmetric VTCs. Data in the region of light pink background are from the asymmetric VTCs. Although the output swing of these VTCs, calculated by *V*_out_/*V*_dd_, may reach 100%, their SNM values are 0 actually, and these data are vertically down shifted for clarity. The light blue area highlights the favorable corner of output swing higher than 95% with SNM larger than 60%. (**B**) First and second gains of the ternary inverters obtained at various *V*_dd_. The blue symbols denote inverters using 2D materials, the green ones are for those based on organic semiconductors, magenta stars are for those with 3D/1D heterojunctions, and the half-filled circles are for those based on silicon-tunneling FETs. The light blue areas highlight the favorable corner of gain larger than 10 with *V*_dd_ less than 5 V. Source of the data are detailed in the figure legend.

A comprehensive summary of the results from various ternary logic devices and circuits identified in the literature is presented in table S1, with performance benchmarked in Fig. 8. While most studies focus on emerging low-dimensional materials (*18*, *23*–*27*, *33*, *35*, *36*, *38*, *39*), results from bulk materials are also included for comparison (*31*, *32*, *34*, *37*, *40*–*47*). High-performance ternary inverters require both full-swing VTC and large SNM, which are essential for constructing more complex circuits. Therefore, in addition to the rail-to-rail VTC, the middle logic state of a ternary inverter should not only have a substantial width but also be positioned near 1/2 *V*_dd_ for optimal operation. Figure 8A compares the output swing and SNM values of ternary inverters from the reference study. Although ternary inverters based on low-dimensional nanomaterials have been successfully implemented, many exhibit asymmetric outputs, and achieving rail-to-rail VTC with meaningful SNM remains rare. However, some inverters based on bulk materials, such as silicon and organic semiconductors (*32*, *40*), show improved performance. Notably, our CNT-SGT–based inverters excel in meeting these criteria, being the only one in the database to achieve full-swing operation with an SNM exceeding 60%. These SNM values are comparable to those reported for high-performance conventional binary CNT inverters (*48*, *49*). It should be noted that the organic semiconductor approach is highly complicated (*32*), and the silicon-based approach (*40*) relies on advanced CMOS processes (130- and 90-nm nodes) to achieve high-performance ternary inverters. Our results suggest that CNTs offer superior performance for MVL applications compared to 2D materials and bulk semiconductors, manifesting the potential of CNT-SGTs. Transition gains are another critical metric for ternary inverters, with lower operating voltage and higher gain being more desirable. Figure 8B compares the first and second transition gains of the ternary inverters across different operating voltages. Most inverters constructed with of 2D materials exhibit gains below 1, whereas both our devices and bulk material-based ternary inverters show gains higher than 10. In addition, our device can achieve high gains at lower voltages, which is particularly advantageous for reducing circuit power consumption. The light blue area in the top left corner of Fig. 8B highlights the region where the operating voltage is less than 5 V and the gain is larger than 10. Only our inverters reach this favorable region, representing the highest gains achieved by ternary inverters constructed with emerging low-dimensional materials. The performance limitations of those inverters have hindered the construction of more advanced ternary logic circuits. To date, NMIN and NMAX (ternary-NAND/NOR) gates have only been fabricated in two reports (*36*, *37*), both using bulk semiconductors. Moreover, an SRAM circuit has been created using 2D black phosphorus/ReS_2_ heterostructures (*38*), although it does not use inverters, limiting its application in general logic circuits. Given these constraints, the prospect for developing more complex circuits with those methodologies appears limited, underscoring the need for alternative strategies to advance the field. In this study, we have successfully developed ternary logic gates (NMIN and NMAX), a ternary 6 T-SRAM, TN, and TNN circuits. These represent the most sophisticated and highest-performing ternary logic circuits ever demonstrated using low-dimensional materials. These fabricated ternary circuits have the same complexity as their binary counterparts while achieving higher information density, highlighting the superiority of ternary logics. The success of these circuits indicates that constructing more complex ternary logic digital circuits with CNT-SGTs is not only feasible but also imminent, marking a substantial advancement of MVL technology. Because 3*^n^* is considerably larger than 2*^n^* as *n* increases, our ternary inverters enable substantially higher bit density, potentially surpassing the fundamental bit limit of traditional binary Boolean logic.

The successes achieved are primarily attributed to the high performance of the ternary inverters, driven by the innovative design of the CNT-SGTs. The ultrathin body and unique energy band structure of CNTs result in a relatively low density of states (*50*), making the energy bands highly sensitive to gate voltage modulation. This facilitates the easy formation of p-n homojunction in our device. In addition, the small effective mass of the symmetrical electrons and holes in CNTs enhance tunneling capabilities, leading to observable NDT/NDR effects, as illustrated in Fig. 1 (B and C). These characteristics highlight the superiority of CNTs over 2D materials for MVL devices. Furthermore, the work function of the bottom gate and the extended source can be engineered to control the NDT effects. These features offer greater flexibility in optimizing CNT-SGT performance, contributing to rail-to-rail VTC and an excellent middle logic level in ternary inverters, simplifying circuit design and fabrication. Although PMOS-style ternary inverters were used for constructing logic gates and circuits, CMOS-style ternary inverters are also feasible, potentially reducing power consumption. In contrast to binary logics, CMOS ternary inverters can be constructed by connecting a p-type CNT-SGT with a conventional n-type CNT transistor, an n-type CNT-SGT with a conventional p-type CNT transistor, or a p-type CNT-SGT with an n-type CNT-SGT, enabling more flexible design for ternary logic circuits. In addition, the CNT-SGT design is adaptable to 2D materials, with NDT phenomena tunable through adjustments of *V*_gs_, *V*_ds_, and the length of the extended source.

Large-scale fabrication is crucial for the development and practical application of MVL VLSI technology. Previous studies on 2D material-based MVL have demonstrated NDT and NDR effects in p-n heterojunctions formed by overlapping distinct 2D semiconducting layers. However, controlling the precise location and size of these overlapped junctions during fabrication has been challenging. While lateral epitaxy growth can yield 2D heterojunctions (*51*), accurately positioning these junctions across a wafer scale remains a major challenge. In contrast, this work introduces a source-gating design for a homojunction, which is feasible for large-scale fabrication and integration. The process is fully compatible with existing CNT-based binary integration processes, enabling seamless incorporation of prior advancements into CNT ternary logic. In addition, hybrid integration of CNT-based binary and ternary circuits is now possible, allowing the most highly scaled parts with complex interconnects in binary logic to be replaced by ternary logic, thereby enhancing information density while simplifying interconnects. Thus, CNT-based MVL technology offers distinct advantages over 2D material approaches in both material properties and fabrication techniques.

Although demonstrated with CNTs in this work, the SGT concept is adaptable to both emerging low-dimensional materials, such as 2D semiconductors and nanowires, and traditional bulk materials, such as organic/oxide semiconductors thin films. This versatility underscores the potential of SGTs in advancing ternary computing architectures.

## MATERIALS AND METHODS

High-purity semiconducting CNT (s-CNT) solutions were prepared by polymer wrapping extraction method using poly[9-(1-octylonoyl)-9H-carbazole-2,7-diyl] and HiPco CNT materials following the procedure reported in our previous work (also see fig. S1) (*52*, *53*). Meanwhile, bottom gate stack (5-/30-nm Ti/Au electrode covered with 5-nm HfO_2_) was fabricated using conventional e-beam lithography (EBL), e-beam evaporation (EBE), lift-off process, and atomic layer deposition on an insulating substrate (Si with 500-nm–thick SiO_2_ was used in this work) (*54*). High-density and uniform s-CNT network films were fabricated by dip-coating method on the substrate with bottom gate stack (*55*). The CNT channels were patterned by EBL, and unwanted s-CNTs outside the channel regions were etched by oxygen plasma using reaction ion etching. Then, source (S) and drain (D) contacts (Ti/Pd/Au with thickness of 0.3/60/20 nm) were fabricated by the same process for the gate electrodes. A 3-nm–thick yttrium film was deposited by EBE and oxidized at 260°C for 30 min on a hotplate in ambient air, which resulted in a thin layer of top oxide Y_2_O_3_ (*56*). Last, another metal layer (40-/50-nm Pd/Au) on the top oxide was fabricated, which partially covered the CNT channel and also connected to the source electrode (fig. S2). The devices and circuits were measured using a probe station (Cascade Summit, 1100), signal generator (Agilent, 33220A), oscilloscope (Agilent, DSO90404A) and semiconductor parameter analyzers (Keithley, 4200). The morphology of the CNT films and fabricated devices were observed by scanning electron microscope.
