# Supplementary material for: High-performance ternary logic circuits and neural networks based on carbon nanotube source-gating transistors
Source: Sci Adv. 2025 Jan 10;11(2):eadt1909. doi: 10.1126/sciadv.adt1909 (PMC11721562; doi:10.1126/sciadv.adt1909)
Supplement: Supplementary file 1 — Supplementary Text Figs. S1 to S11 Table S1 [file sciadv.adt1909_sm.pdf]

Science Advances
AAAS

Supplementary Materials for

High-performance ternary logic circuits and neural networks based on carbon nanotube source-gating transistors

Xuehao Zhu *et al.*

Corresponding author: Panpan Zhang, tanji_ic@bupt.edu.cn; Yu Cao, yu_cao@pku.edu.cn; Qiliang Li, qiliang.li@pku.edu.cn; Xuelei Liang, liangxl@pku.edu.cn

Sci. Adv. **11**, eadt1909 (2025)
DOI: 10.1126/sciadv.adt1909

This PDF file includes:

Supplementary Text
Figs. S1 to S11
Table S1

Supplementary Text

TCAD simulation of the working mechanism of the CNT-SGT

The TCAD model is implemented using Sentaurus. The constant mobility model is turned on to eliminate the impact of mobility degradation on the drain current, accompanied by the feature of high field saturation. Note that the Hurkx tunneling model is invoked to calculate the band-to-band tunneling branch at the homo-junction within the channel before the device fully turns on. Geometrical and material parameters fed into the model are listed below. Systematic simulations reveal that two essential conditions must be fulfilled for the onset of the NDT phenomenon: (1) Enough band misalignment to induce a p-n homo-junction within the channel; (2) the available states to accommodate the tunneled electrons at the n-side varying with the gate bias V_{gs}.

Material and structure parameters for simulation are listed below

Geometrical Parameters		Material Parameters	
Parameter	Value	Parameter	Value
Contact length	1 μm	Density of states (N_C)	3×10^{17} $cm^{-3}eV^{-1}$
Channel length	3 μm	Density of states (N_V)	3×10^{17} $cm^{-3}eV^{-1}$
Length of extended source	From 1.5 μm to 2.5 μm	Band gap of the CNT film	0.55 eV
Thickness of the CNT film	3 nm	Electron affinity of the CNT film	4.0 eV
Thickness of the bottom HfO_2 layer	15 nm	Barrier tunneling mass	0.1 m_0, 0.3 m_0
Thickness of the top Y_2O_3 layer	5 nm	Relative Permittivity of HfO_2	19

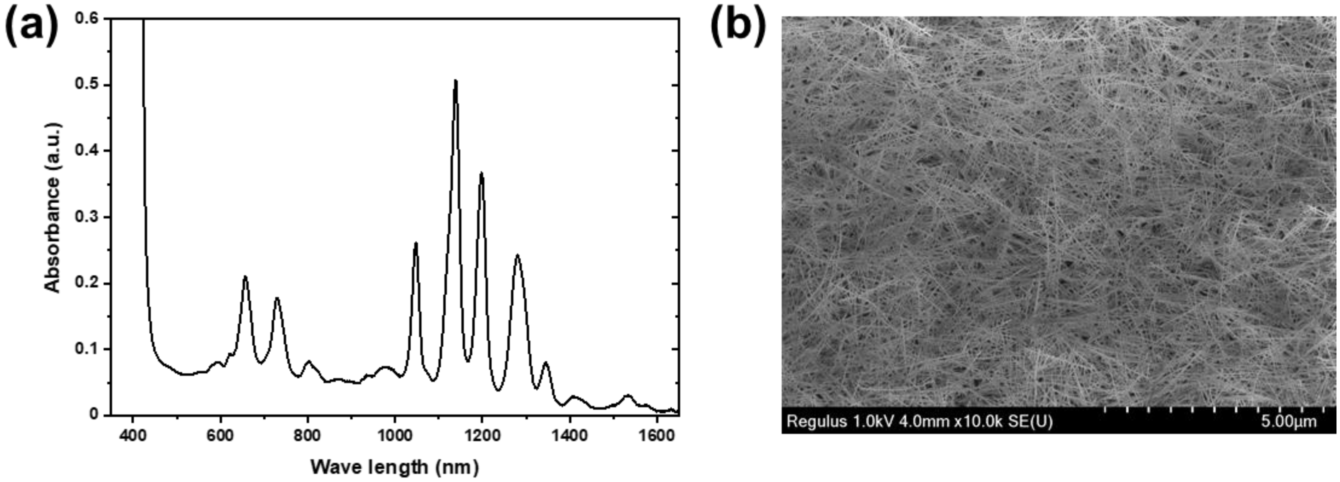

Fig. S1. Characterization of CNT material.

(a) Absorption spectrum of the high purity semiconducting HiPco CNT solution used in this work. (b) SEM image of the high density and uniform CNT film for device fabrication.

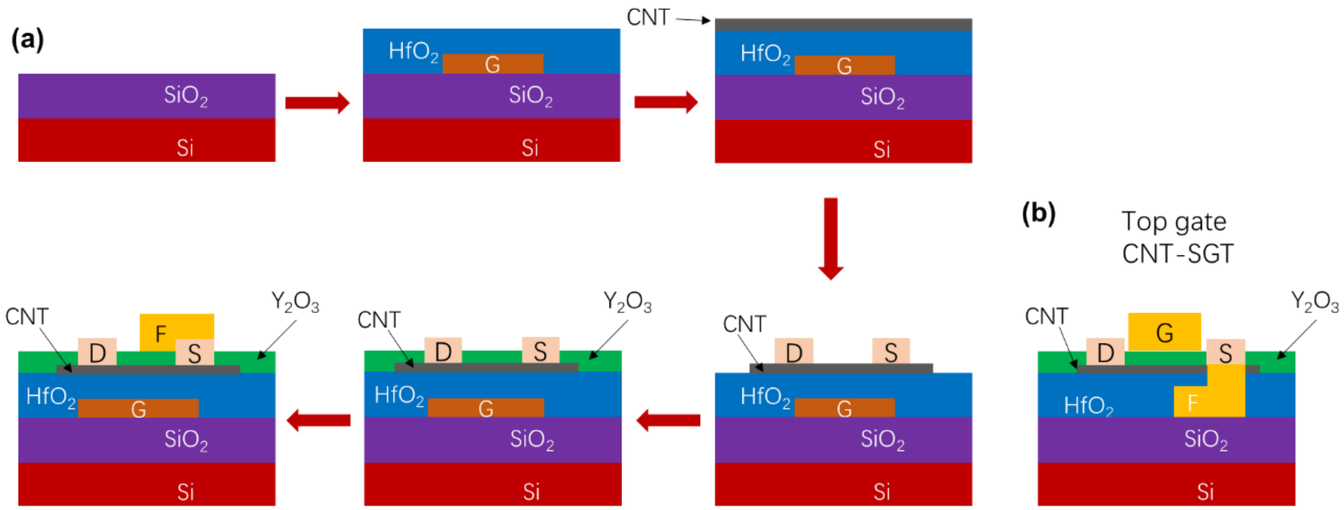

Fig. S2. Device structure and fabrication process of CNT-SGTs.
(a) Fabrication process flow of the bottom gate CNT-SGTs. (b) Structure of top gate CNT-SGT.

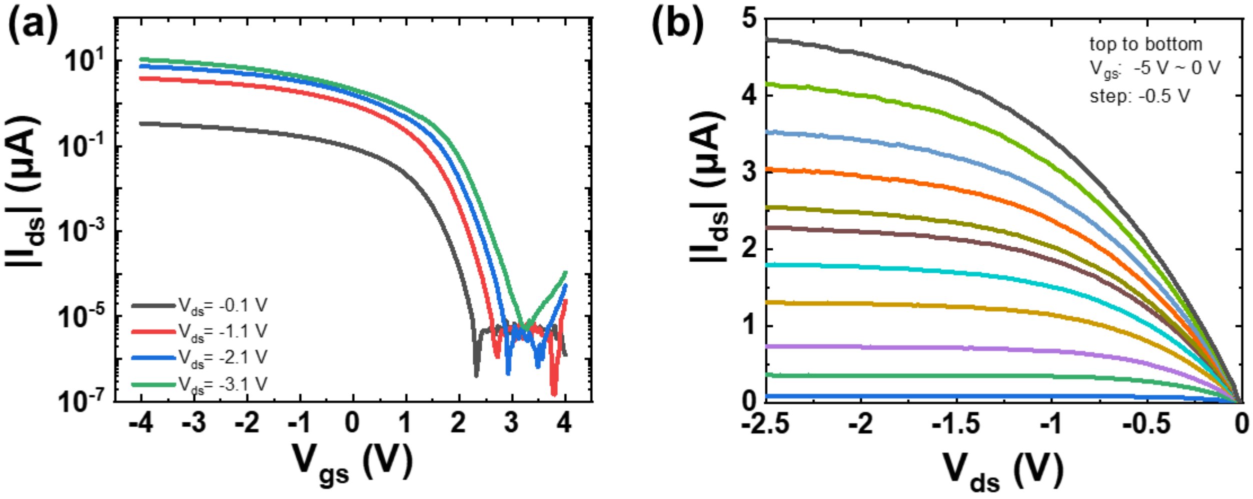

Fig. S3. Electrical characterization of conventional CNT transistor.

(a) Transfer and (b) output curves of a conventional CNT transistor with W=10 μm and L=5 μm as a comparison of the CNT-SGTs in the main text.

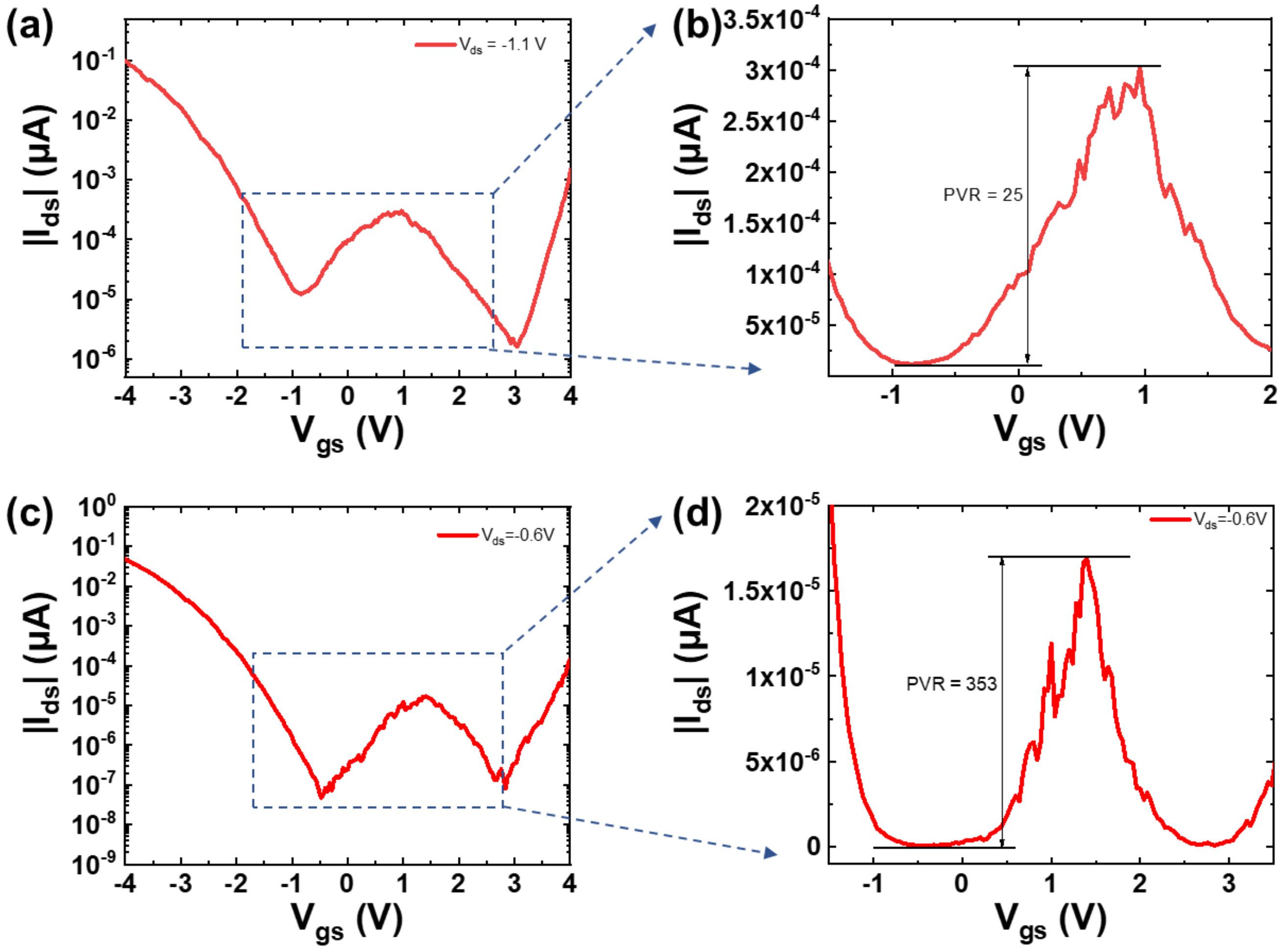

Fig. S4. The determination of PVRs of two CNT-SGTs.

(a) and (c) are the transfer curves in logarithm scale, and they are also ploted in linear scale as shown in (b) and (d) respectively to facilitate the extraction of PVR values.

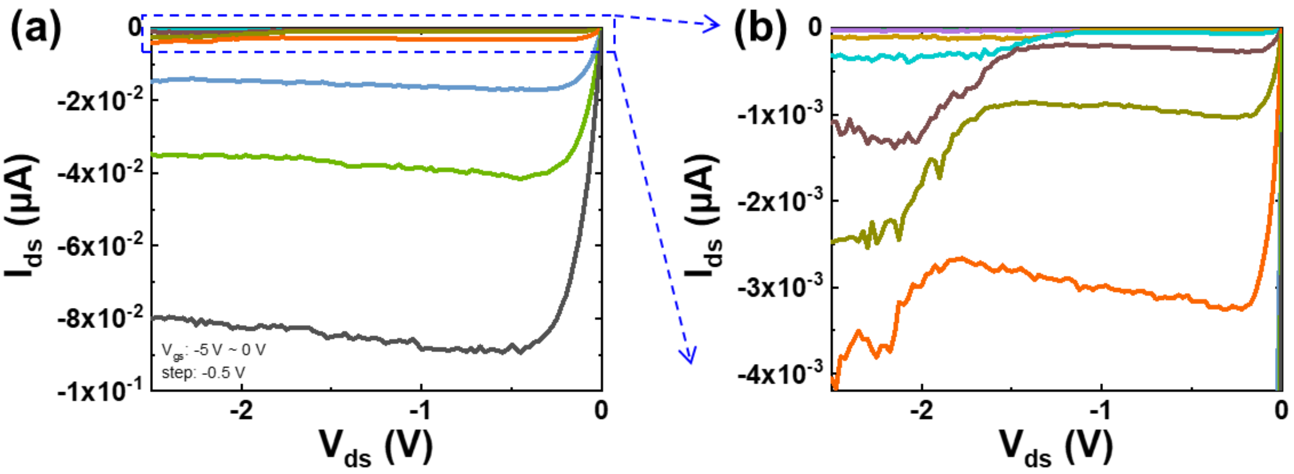

Fig. S5. NDR effects in the output curves of CNT-SGT.

(a) Output curves of the device in Fig. 1 of the main text plotted in linear scale. (b) Zoom-in plot of the low current curves in (a).

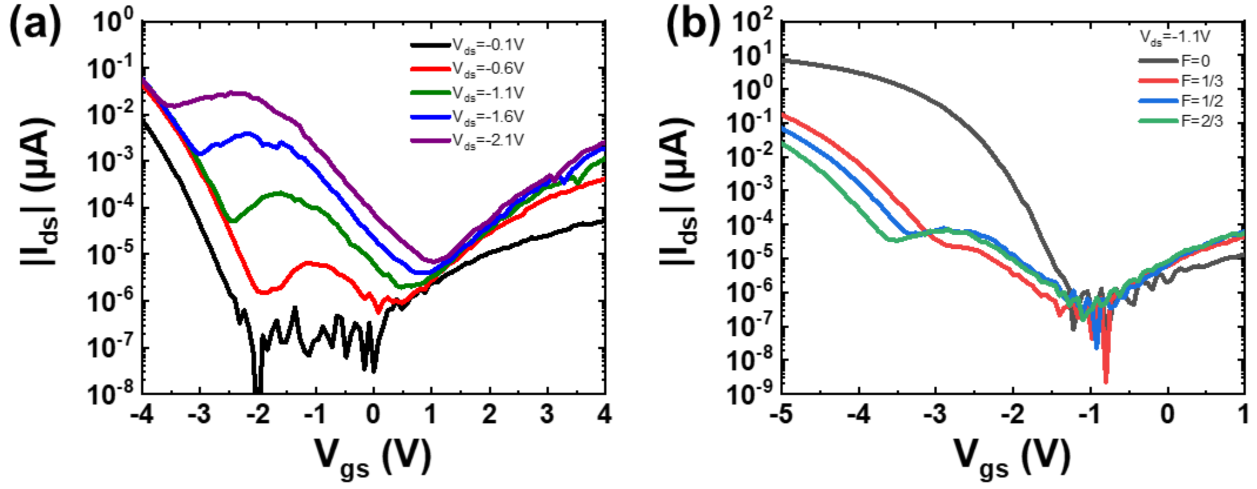

Fig. S6. Transfer curves of more typical CNT-SGTs.

(a) W/L = 10/5 μm, F = 2/3 and (b) W/L = 10/5 μm, respectively.

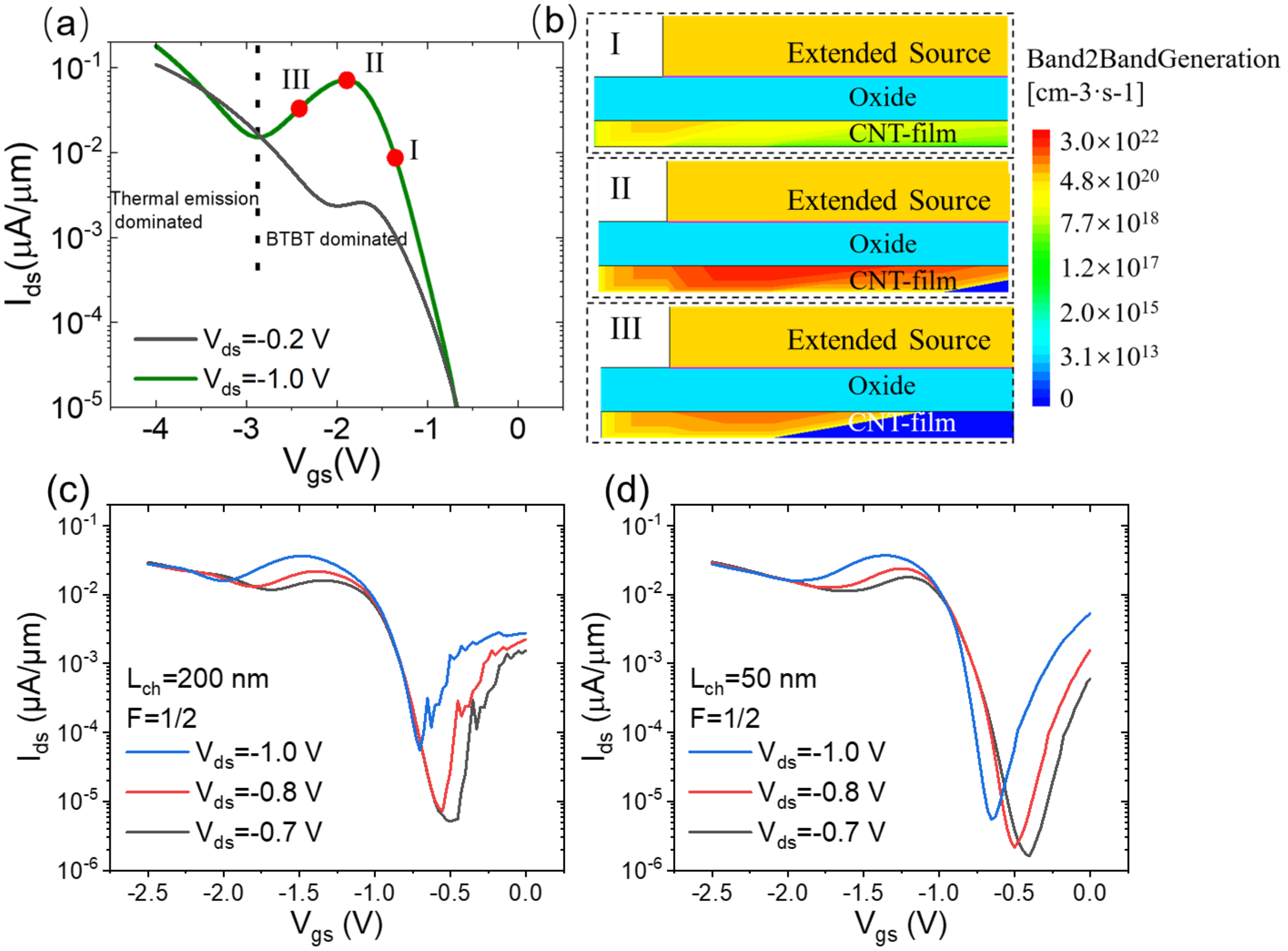

Fig. S7. NDT effects confirmed by TCAD simulations.

(a) Simulated transfer curve of CNT-SGT of L=3μm and F=1/2. (b) The generation rate of the conductance band electrons at the homo-junction within the channel due to the BTBT mechanism under different gate biases. (c) and (d) are simulated transfer curves of CNT-SGT of L= 200 nm and 50 nm, respectively.

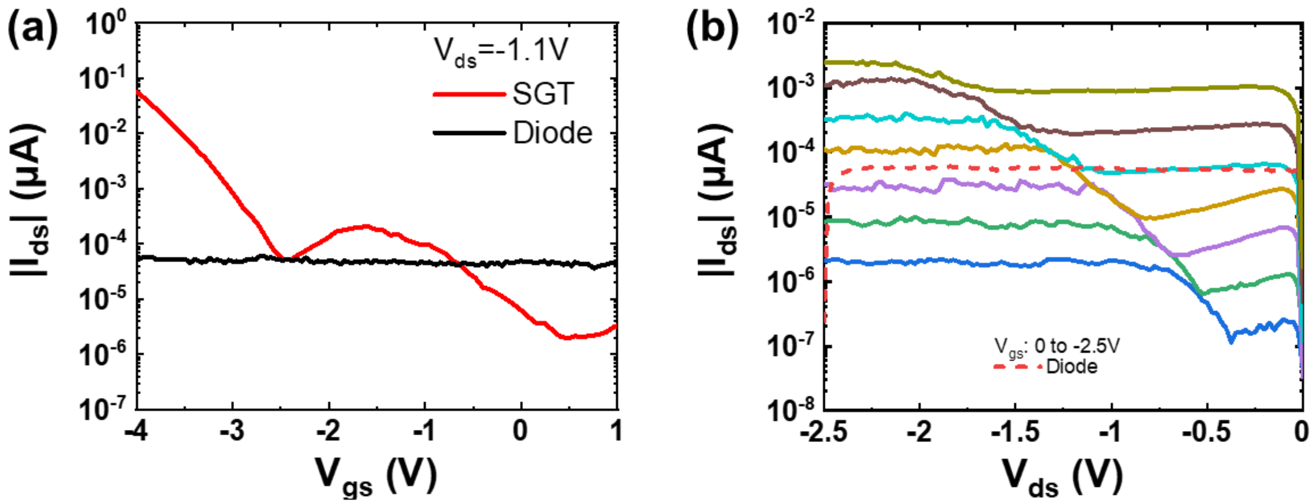

Fig. S8. Explanation for the behavior of ternary inverter.

(a) Typical transfer characteristics of the CNT-SGT and load transistor in the ternary inverters, showing the impedance matching feature of the two components. (b) Corresponding output load lines. Both of (a) and (b) demonstrate three distinct logic states achieved in the ternary inverter.

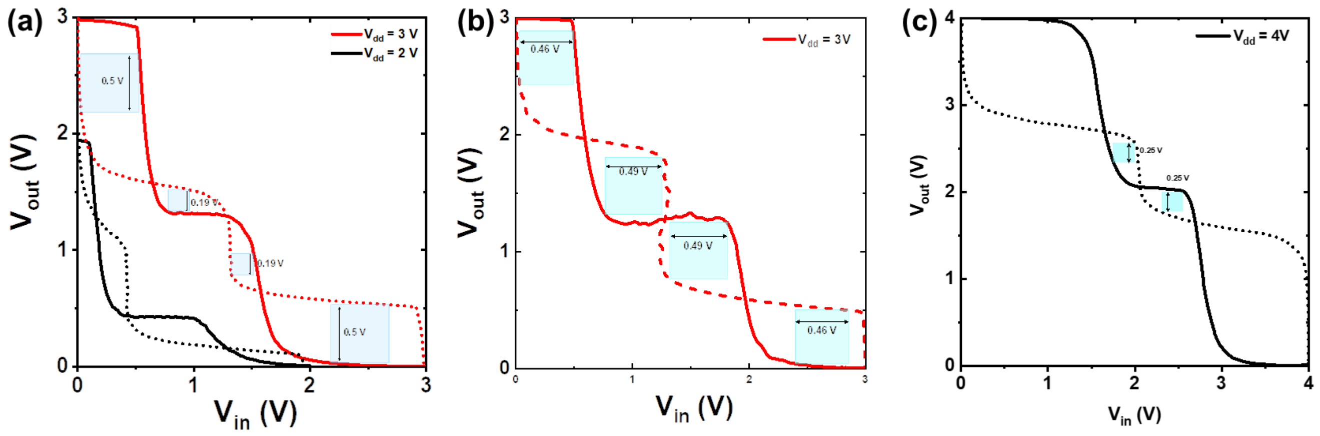

Fig. S9. Typical VTCs and SNMs of more ternary inverters.

(a) The SNM of the inverter shown in Fig. 4(a to c) of the main text. (b) SNM of 0.46 V for a ternary inverter, corresponding to 61.2% of the ideal values. (c) SNM of 0.25 V.

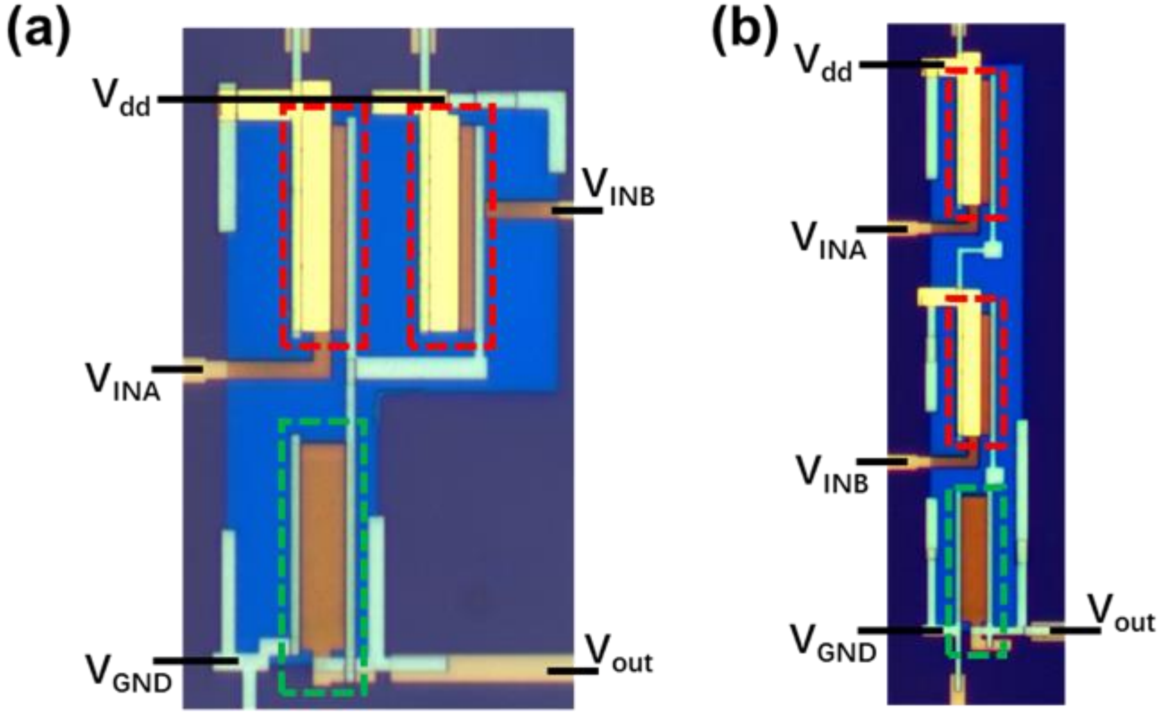

Fig. S10. Optical images of the fabricated logic circuis.
(a) NMIN circuits. (b) NMAX circuits.

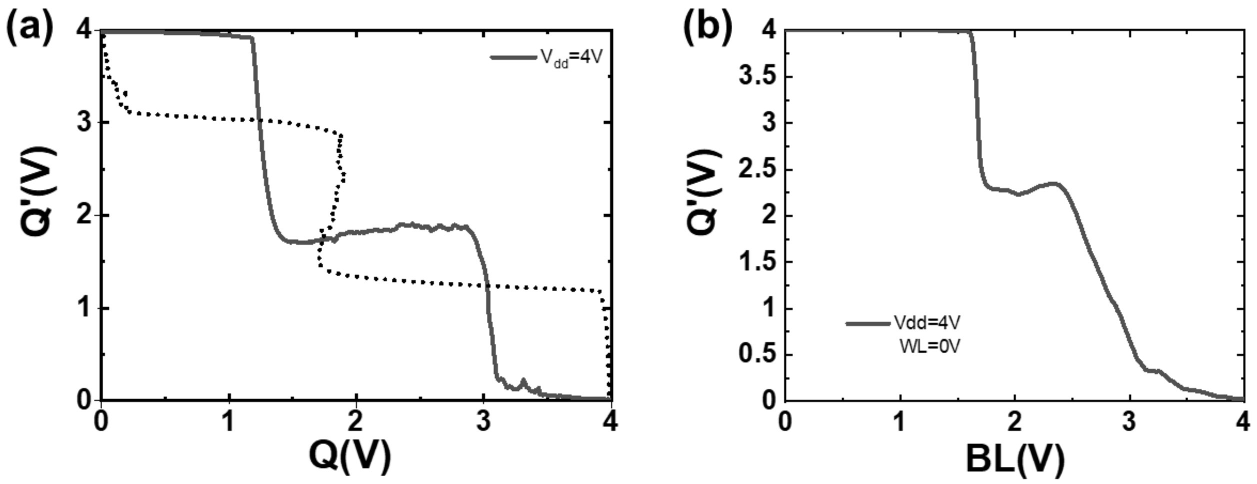

Fig. S11. Read and write margins of another ternary SRAM.

(a) Read margins. (b) Write margins.

Table S1. Performance benchmarks of reported ternary inverters and circuits.

Material	Structure	Working principle	PVR	Operating voltage			Rail-to-rail of inverter	Output swing[b)]	Gain1/ Gain2	SNM of inverter[c)]	Circuits	Logic level deviation of circuits $\Delta V/V_{dd}$	Ref.
				V_{dd} (V)	V_{in} (V)	V_{out} (V)							
CNT Network	1D Homo-junction	NDT	10-300 Max: 353	2	(0, 2)	(0, 1.95)	√	97.5%	12.5/2.5	36%	Inverter/ NMIN/ NMAX/ S-RAM	NMIN: 2.09%; NMAX: 2.78% (For all 3 levels) NMIN: 4.92%; NMAX: 5.83% (For level '1' only)	This work
				3	(0, 3)	(0, 3)	√	100%	15/6	61.2%			
				4	(0, 4)	(0, 4)	√	100%	15/25	61%			
BP@ReS_2	2D vdW Hetero-junction	NDR	16	1.8	(0.7, 1.5)	(0.7, 1.5)	×	47.1%	-	-	S-RAM	-	(*38*)
MoS_2@$MoTe_2$	2D vdW Hetero-junction	NDT	~50	1	(-40, 40)	(0, 1)	×	100%	0.004/0.004	-	Inverter		(*39*)
				2	(-40, 40)	(0, 2)	×	100%	0.008/0.016				
MoS_2@BP	2D vdW Hetero-junction	NDT	~50	1	(-1, 1)	(0, 1)	×	100%	-	-	Inverter	-	(*35*)
				2	(-1, 1)	(0.2, 2)	×	90%	12/8	-			
				3	(-1.5, 1)	(0.5, 3)	×	83%		-			
				4	(-1.5, 1)	(0.8, 4)	×	80%	-	-			
BP@ReS_2	2D vdW Hetero-junction	NDR	3	2	(5, 25)	(0.2, 1.75)	×	77.5%	0.197/0.465	-	Inverter	-	(*33*)
MoS_2@WSe_2	2D vdW Hetero-junction	NDT	~2000	1	(0, 1)	(0.15, 0.9)	×	75%	4.5/2.5	8%	Inverter	-	(*23*)
MoS_2@$MoTe_2$	2D vdW Hetero-junction	ZDT[a)]	-	2~6	(-15,30)	(0, V_{dd})	×	100%	0.65/1.7	-	Inverter	-	(*18*)
h-BN@$MoTe_2$	2D vdW Hetero-junction	NDT	-	2	(-60, 60)	(0, 2)	×	100%	0.16/0.01	-	Inverter	-	(*24*)
MoS_2@$MoTe_2$	2D vdW Hetero-junction	NDT	-	2	(-20, 5)	(0, 2)	×	100%	1.2/0.35	-	Inverter	-	(*25*)
MoS_2@$MoTe_2$	2D vdW Hetero-junction	NDT	1000	26	(-60,10)	(10, 26)	×	61.5%	14.5/21	-	Inverter	-	(*26*)
Graphene/WSe_2	2D vdW Hetero-junction	NDT	-	4	(-30, 30)	(0, 4)	×	100%	0.42/1.26	-	Inverter	-	(*27*)
ZnO@Al4MP	2D Superlattice	ZDT	-	5	(0, 5)	(0.1, 4.75)	√	93%	5/2.9	24%	Inverter/ NMIN/ NMAX	NMIN: 6.89%; NMAX: 6.56% (For all 3 levels) NMIN: 7.33%; NMAX: 6.67% (For level '1' only)	(*36*)
Si	3D Tunnelling-FET	ZDT	-	0.5	(0, 0.5)	(0.05, 0.5)	√	90%	2.9/1.9	20.8%	Inverter/ Latch	-	(*40*)
				1	(0, 1)	(0, 1)	√	100%	4.2/3.3	45.2%			
Organic Semiconductors	3D Hetero-junctions	NDT		8	(0,8)	(0,8)	×	100%	15/30	48%	Inverter		(*41*)
Organic Semiconductors	3D Printed Vertical FET	ZDT	-	1	(0, 1)	(0.05, 0.95)	√	90%	3.25/3.5	24%	Inverter/ NMIN/ NMAX	NMIN: 6.11%; NMAX: 5% (For all 3 levels)	(*37*)

												NMIN: 10%;<br>NMAX: 8.33%<br>(For level ‘1’ only)	
Organic Semiconductors	3D Vertical tandem two-channel series	NDT	~100	5	(0, 5)	(0, 5)	√	100%	25/5	0%	Inverter	-	(*31*)
Organic Semiconductor	3D Hetero-junction	NDT	-	6	(0, 6)	(0, 6)	√	100%	35/50	58.8%	Inverter	-	(*32*)
Organic Semiconductors	3D Hetero-junction	NDT	3	50	(0, 50)	(0, 50)	√	100%	17/23	0%	Inverter	-	(*42*)
Organic Semiconductors	3D Anti-ambipolar Transistors	NDT	~1000	10	(0, 4)	(0, 8)	×	80%	15/12	-	Inverter	-	(*43*)
Cu_2O@IGZO	3D Hetero-junction	NDT	45	5	(-10, 10)	(0, 4.5)	×	90%	-	-	Inverter	-	(*44*)
				20	(-5, 25)	(0, 20)	×	100%	2/7	-			
ReS_2@IGZO	3D&2D Two-channel series	ZDT	-	1	(-5, 5)	(0, 0.9)	×	90%	1.2/2.6	-	Inverter/ Frequency doubler	-	(*45*)
Organic@$MoTe_2$	3D&2D Heterojunction	NDR	137	-1	(0, 1.75)	(-0.1, -0.8)	×	70%	0.82/1.27	-	Inverter	-	(*46*)
In_2O_3@CNT	3D&1D Printed Heterojunction	NDT	~1000	2	(-0.5, 2)	(0, 2)	×	100%	4.92/5	-	Inverter		(*47*)
In_2O_3@CNT	3D&1D Printed Heterojunction	NDT	~10	1	(0, 3)	(0, 1)	×	100%	2/1	-	Inverter	-	(*34*)
				2	(0, 3)	(0, 2)	×	100%	5/3	-			
				3	(0, 3)	(0, 3)	√	100%	5/12	0%			

[a)]The ZDT working principle of the ternary inverters and circuits stands for zero differential transconductance according to Ref. (*12*).
[b)]The output swing is calculated by V_{out}/V_{dd}, which may reach 100% for asymmetric VTCs, but the corresponding SNM value is 0 actually.
[c)]The SNM of inverters are extracted from the corresponding references.
